# Screening Impact of Anti-HDV Reflex Testing Among HBsAg-Positive Individuals

**DOI:** 10.3390/jcm15114019

**Published:** 2026-05-22

**Authors:** Tor Regev-Sadeh, Ziv Neeman, Naama Schwartz, Orit Rozenberg, Fadi Abu Baker, Tarek Saadi, Mifleh Tatour, Rawi Hazzan

**Affiliations:** 1 Bruce Rappaport Faculty of Medicine, Technion-Israel Institute of Technology, Haifa 3525433, Israel; 2 Department of Radiology, Emek Medical Center, Afula 1834111, Israel; 3 Emek Medical Center Laboratories, Clalit Health Services, Afula 1834111, Israel; 4 Department of Gastroenterology and Hepatology, Hillel Yaffe Medical Center, Hadera 38100, Israel; 5 Liver Unit, Rambam Health Care Campus, Haifa 3109601, Israel; 6 Department of Gastroenterology, Rambam Health Care Campus, Haifa 3109601, Israel; 7 Clalit Health Services, Northern Region, Nazareth 1622222, Israel; 8 Azrieli Faculty of Medicine, Bar-Ilan University Henrietta, Safed 1311502, Israel

**Keywords:** hepatitis D virus, hepatitis B surface antigen, reflex testing, HDV screening, HBV–HDV coinfection, population-based screening

## Abstract

**Background**: Hepatitis D virus (HDV) causes one of the most severe forms of chronic viral hepatitis. Despite its severity, universal screening of hepatitis B surface antigen (HBsAg)-positive individuals, as recommended by European guidelines, is not widely implemented. This study aimed to evaluate the yield of reflex HDV testing and to characterize HBV carriers who tested positive or negative for anti-HDV. **Methods**: A retrospective cohort study was conducted using the Clalit Health Services database in northern Israel (2014–2024). HBsAg-positive patients were categorized into two groups: those screened for HDV via reflex testing (2019–2024) and those tested based on clinical discretion (2014–2019). We compared these cohorts to evaluate the impact of reflex screening on coverage, diagnostic yield, and time to diagnosis. **Results**: Among 1336 HBsAg-positive individuals, HDV screening rates increased from 57.5% to 93.1% following reflex implementation. HDV seropositivity increased from 3.17% to 6.48% (*p* = 0.02). Ethiopian-born individuals had significantly higher positivity than others (10.4% vs. 3.9%, *p* = 0.0221). The average time from HBV diagnosis to HDV testing decreased from 38.1 ± 31 months (median 37.5) to 1.3 ± 6.1 months (median 0). **Conclusions**: Anti-HDV reflex testing significantly improved screening coverage, increased detection of anti-HDV seropositive cases and was associated with shorter time to serologic identification. These findings support the integration of reflex testing into national screening policies to enable earlier diagnosis and reduce the burden of infection.

## 1. Introduction

Hepatitis D virus (HDV) is a single-stranded RNA virus that requires the presence of hepatitis B virus (HBV) for replication. HDV infection occurs either as coinfection with HBV or as a superinfection in individuals with pre-existing chronic HBV infection, and these two entities differ substantially in their clinical course. Coinfection typically presents as acute hepatitis and may resolve spontaneously, whereas superinfection is associated with progression to chronic infection in approximately 80% of cases [1,2,3]. Compared with isolated HBV infection, HDV is associated with a 2–3-fold higher risk of cirrhosis and/or hepatocellular carcinoma, as well as more rapid progression to advanced liver disease, characterized by accelerated fibrosis progression and earlier development of cirrhosis, which may occur within 5–10 years of infection in a substantial proportion of patients, compared with a typically slower course in HBV monoinfection [2,4,5]. This more aggressive disease course is further reflected in an approximately 2-fold higher risk of hepatic decompensation and liver-related mortality compared with HBV monoinfection [5,6]. From an epidemiological perspective, HDV infection is estimated to affect approximately 5% of individuals with chronic HBV infection globally, corresponding to 12–20 million people worldwide, with substantial geographic variability and a high degree of underdiagnosis [3,5].

Given the severity of HDV infection, universal screening is essential, yet guidelines for HDV testing vary across organizations. The American Association for the Study of Liver Diseases (AASLD) prioritizes screening individuals at elevated risk of HDV infection, including those originating from HDV-endemic regions and individuals with known risk factors such as injection drug use or HIV coinfection [5]. In contrast, the 2023 European Association for the Study of the Liver (EASL) guidelines recommend anti-HDV screening for all patients who test positive for HBsAg [3]. Universal screening can facilitate early diagnosis, help to prevent further transmission and initiate timely treatment [7]. A number of studies have shown that risk-based screening alone is insufficient, consistently failing to identify a significant proportion of HDV carriers [8,9,10,11]. Despite these updated recommendations, many HBV carriers worldwide remain untested for HDV, including those in our hospitals and community clinics [12,13]. This gap in screening is often attributed to concerns regarding cost-effectiveness, limiting broader adoption in clinical practice.

This study aimed to evaluate the impact of anti-HDV reflex testing on screening coverage, diagnostic yield, and time to serologic identification, and to characterize the demographic and clinical profile of HBsAg-positive individuals who tested positive or negative for anti-HDV in northern Israel. These findings may contribute to a better understanding of the clinical effectiveness and potential cost-efficiency of systematic HDV screening strategies.

## 2. Materials and Methods

Our study is a large retrospective analysis that included all patients who tested positive for HBsAg between May 2014 and May 2024 in the central laboratory of the Northern District of the “Clalit” registry, encompassing a population of approximately 500,000 Israeli citizens. The study adhered to the ethical principles outlined in the Declaration of Helsinki and received approval from the local institutional review board (approval number: EMC-0173-23, approval date: 21 March 2024). All data were anonymized to ensure patient confidentiality and used solely for research purposes. Data were extracted using the MDClone platform version 11.2 (http://www.mdclone.com, accessed on 20 May 2026), a big data digital database that integrates information from the patient’s entire medical records which integrates longitudinal electronic medical records from community clinics, laboratories, emergency departments, and hospitals. The extracted variables included demographics such as age at HBV diagnosis, sex, country of birth, socioeconomic status; background diagnoses and comorbidities including diabetes mellitus, hypertension, ischemic heart disease, chronic kidney disease, and HIV; and laboratory parameters including ALT, AST, albumin, bilirubin, INR, platelets, and creatinine, in addition to HBV and HDV test results.

All diagnosed HBV patients, referred to as HBsAg-positive, at our institution underwent anti-HDV reflex testing between May 2019 and May 2024 as part of a universal screening program, forming the reflex cohort. Due to budgetary constraints, the second screening stage, polymerase chain reaction (PCR) confirmation, was not incorporated into the reflex screening protocol. Instead, patients who tested positive for anti-HDV were referred to perform PCR testing through standard care pathways. Follow-up data on these confirmatory tests were incomplete, as some patients may not have undergone testing or may have completed it in external laboratories that were not linked to our system. Therefore, the PCR results were excluded from the current analysis. To evaluate the impact of universal screening, we compared the reflex cohort to HBV-positive patients identified between May 2014 and May 2019, before the implementation of routine HDV reflex testing. During this earlier period, HDV testing was conducted selectively based on clinical suspicion, and these patients constituted the pre-reflex cohort. Patients with any record of an anti-HDV test or HDV PCR test prior to being diagnosed with HBsAg positivity were excluded from the study.

Anti-HDV antibodies were analyzed with an indirect chemiluminescence immunoassay (CLIA) on the Liaison XL murex Anti-HDV kit (DiaSorin, Saluggia, Italy). Magnetic particles were coated with biotinylated recombinant HDAg and mouse IgG anti human isoluminol–antibody conjugate. Hepatitis B surface antigen (HBsAg) in human serum was analyzed with the electrochemiluminescence immunoassay “ECLIA” kit on the Cobas e601/e801 analyzers (Roche Diagnostics GmbH, Mannheim Germany). A sandwich complex of monoclonal and polyclonal anti-HBsAg antibodies labeled with ruthenium were used, and streptavidin-coated microparticles had a reaction based in the interaction of biotin and streptavidin.

To assess the diagnostic yield of universal reflex anti-HDV screening, we compared the proportion of anti-HDV-positive cases among HBsAg-positive individuals between the pre-reflex and reflex cohorts. Screening coverage and anti-HDV positivity rates were compared between the pre-reflex and reflex periods. Clinical and demographic characteristics were compared between patients with positive and negative anti-HDV results among those tested. Time to serologic identification was calculated as the interval in months between the first documented HBsAg-positive result and the date of anti-HDV testing and was compared between the pre-reflex and reflex cohorts. Categorical variables were compared using the Chi-square test or Fisher’s exact test, and continuous variables were compared using Student’s *t*-test or the Wilcoxon rank-sum test. A stepwise logistic regression model was used to evaluate factors associated with anti-HDV seropositivity among tested individuals. Statistical analysis and data management were performed using SAS Enterprise Guide version 8.3 (SAS Institute Inc., Cary, NC, USA). All tests were two-sided, and a *p*-value < 0.05 was considered statistically significant. This study is reported in accordance with the STROBE guidelines for observational studies.

## 3. Results

A total of 147,267 individuals were tested for HBV between 2014 and 2024, of whom 1662 (1.1%) were HBsAg-positive. Of these, 1336 (80.4%) met the inclusion criteria and were included in the analysis, comprising a total of 988 patients from the pre-reflex period and 348 from the reflex period. Table 1 summarizes the baseline demographic, comorbidity, and biochemical characteristics of the 39 patients who tested positive for HDV, divided by study period. Both groups were generally similar, except for a higher proportion of Ethiopian-born individuals.

**Figure 1:**
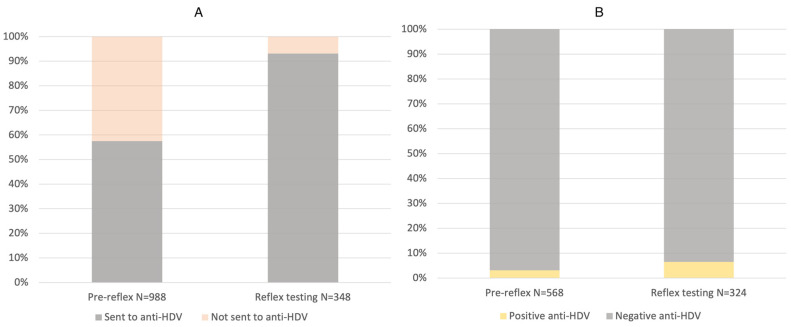
Distribution of HDV testing and test outcomes among HBsAg-positive patients stratified before and during reflex testing implementation. (**A**) Patients tested versus not tested for anti-HDV in each period. (**B**) Among patients who performed anti-HDV test, positive and negative anti-HDV results in each period.

Figure 1 presents the proportion of HBsAg-positive patients who were tested for HDV and their corresponding test outcomes in each period. Among HBsAg-positive individuals, the proportion tested for HDV increased from 57.5% in the pre-reflex period to 93.1% in the reflex period, as shown in Figure 1A. HDV positivity among those tested increased from 3.17% to 6.48% (Chi-square test, *p* = 0.02), as shown in Figure 1B.

Table 2 demonstrates characteristics of patients that underwent anti-HDV reflex testing, stratified by their test results. The groups were similar in age, gender, socioeconomic status, and comorbidities. Two factors were statistically significant in the univariate analysis: diabetes mellitus diagnosis (more frequent in patients with negative HDV compared with positive HDV; 15.9% vs. 2.6% respectively; *p* = 0.0234), and the testing period (more frequent positive HDV in the reflex period compared to the standard testing period; 6.5% vs. 3.2% respectively; *p* = 0.0200). Stepwise multivariable logistics, which initially included diabetes mellitus and a testing period, resulted in only the period factor remaining in the final model. Patients screened during the reflex testing period had 2.1 times higher odds of testing positive for anti-HDV antibodies compared to those tested in the standard testing period (OR 2.1, 95%CI: 1.11–4.02).

**Figure 2:**
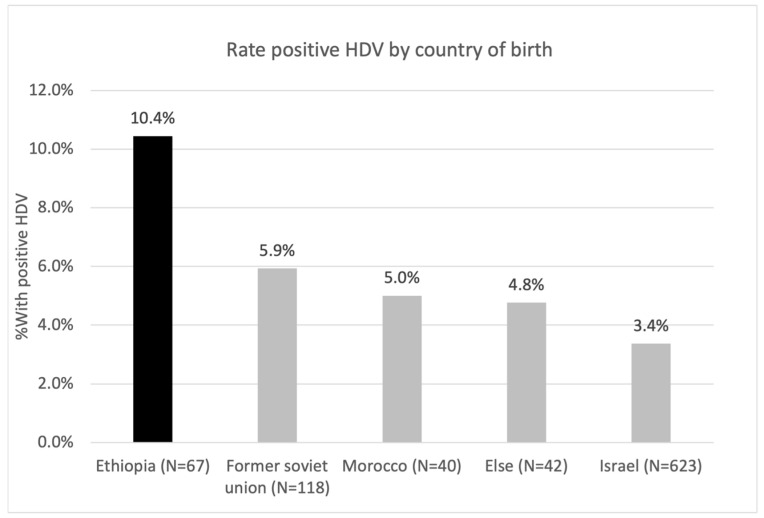
Anti-HDV positivity rate among HBV-positive individuals by country of birth (May 2014–May 2024, Northern District).

Although country of birth was not statistically significant, the HDV-positive percentage clearly highlights the difference between Ethiopian-born patients and the rest of the patients (Figure 2). When comparing Ethiopian-born individuals to all other groups combined, the proportion of HDV-positive cases was significantly higher (10.4% vs. 3.9%; *p* = 0.0221).

**Figure 3:**
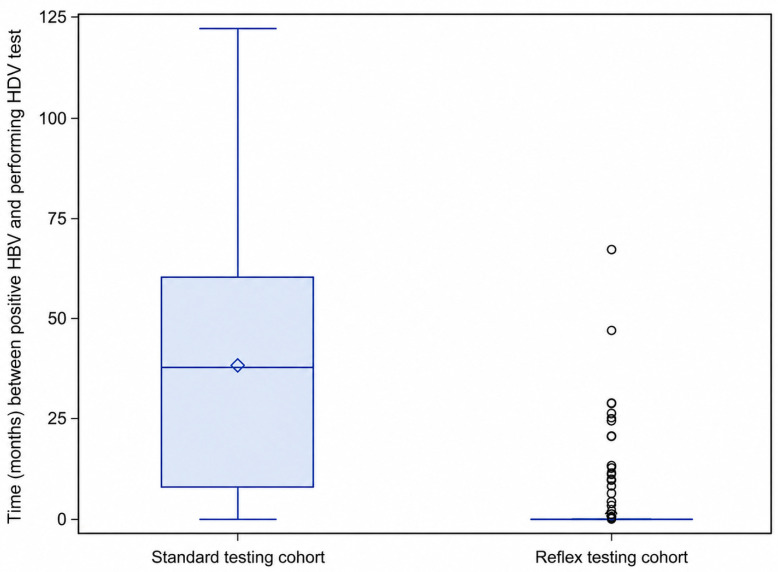
Time interval (months) between a positive HBV result and subsequent HDV testing.

Figure 3 illustrates the time interval (in months) between a positive HBV result and subsequent HDV testing. In the pre-reflex cohort, the median interval was 37.5 months (IQR: 7.5–60.3 months), compared to 0 months in the reflex testing cohort (IQR: 0–0 months). The mean intervals were 38.1 ± 31 months and 1.3 ± 6.1 months, respectively.

## 4. Discussion

In May 2019, HaEmek Medical Center, in collaboration with Clalit Health Services’ Northern District, became the first institution in Israel to implement anti-HDV reflex testing as part of a universal screening strategy. To the best of our knowledge, this is the first published study to provide comprehensive data on anti-HDV reflex screening in Israel, thereby addressing a gap in the global and national literature. The distinct contribution of our study lies in its dual value: it offers real-world epidemiological data on HDV among HBsAg-positive individuals and presents practical insights into the clinical implications and feasibility of integrating reflex testing within a large population-based healthcare system.

Following the implementation of reflex screening, we observed an increase in anti-HDV positivity from 3.17% to 6.48% and a rise in screening coverage from 57.5% to 93.1% among HBsAg-positive individuals. Although positivity rates varied across studies, our findings are consistent with the overall European trend of an increased anti-HDV screening yield following the implementation of reflex testing. Parfut et al. (France) reported a rise in anti-HDV positivity from 6.8% to 10.3% following the implementation of reflex testing, with HDV-RNA detected in 46.7–100% of those who tested positive. Their study demonstrated that reflex testing more than doubled the number of HDV diagnoses [14]. Bernhard et al. (Austria) found that reflex anti-HDV testing increased screening from 65.1% to 98.0%. Anti-HDV positivity was 9.3% in the reflex cohort vs. 5.3% in the standard cohort, with HDV-RNA detectable in 76.2% of positive cases [15]. Cossiga et al. (Italy) reported that reflex testing raised screening coverage from 16.4% to 100%. Although the anti-HDV positivity rate decreased (16.6% to 10.7%), the absolute number of positive cases increased (14 to 52), with HDV-RNA detectable in 53% of tested patients [11]. Palom et al. (Spain) found that anti-HDV testing rates increased from 7.6% to 93% after reflex testing, with primary care rising from 2% to 100% and academic hospitals from 23% to 91%. Anti-HDV positivity remained similar (9.6% vs. 8.1%), but more cases were identified. HDV-RNA was detectable in 65% of positives [8]. Although our analysis was limited to antibody detection, our data, alongside these recent studies, highlight the potential of reflex testing to improve screening uptake, facilitate earlier HDV diagnosis, and support more timely clinical intervention.

In our study, anti-HDV screening was conducted much earlier in the reflex cohort, with a median delay of 0 months from HBsAg diagnosis, compared to 37.5 months in the pre-reflex cohort. This reduction in time to testing enhances opportunities for earlier clinical intervention, monitoring, and evaluation for emerging antiviral therapies such as bulevirtide, all of which contribute to improved long-term outcomes. A recent study by Weichselbaum et al. highlights that early diagnosis, combined with appropriate follow-up and treatment, may prevent long-term complications, including progression to cirrhosis, hepatic decompensation with its associated morbidity and mortality, and hepatocellular carcinoma. Their findings further suggest that early intervention may promote regression of liver fibrosis and cirrhosis in some patients. These findings emphasize that timely HDV screening is not only diagnostic but also preventive in nature [7].

A subanalysis by country of birth revealed important demographic insights. Anti-HDV positivity was higher among individuals born in Ethiopia, the former Soviet Union, and Morocco. When examining the proportion of anti-HDV-positive cases among HBsAg-positive individuals, we found a statistically significant association with being born in Ethiopia, compared to individuals born elsewhere. A previous study we conducted between 1998 and 2016 showed a similar trend among individuals born in Ethiopia [13]. Notably, six of seven Ethiopian-born HDV-positive patients were identified during the reflex screening period. Despite this known endemicity, risk-based testing has overlooked high-risk individuals. These findings stress the need for structured screening protocols.

Interestingly, diabetes mellitus was less prevalent among anti-HDV-positive patients, at 2.6%, compared to 15.9% in HBV mono-infection patients. A similar observation was reported by John et al., with diabetes prevalence of 20.3% and 30.3%, respectively [16]. This contrasts with the general association between HBV infection and higher DM prevalence compared to uninfected populations, as suggested by other studies [17,18]. These findings in co-infected individuals may reflect a distinct clinical or biological profile. Though the underlying mechanisms remain unclear and require further investigation.

This study has several limitations. First, while reflex anti-HDV testing was broadly implemented, confirmatory HDV-RNA PCR testing was not included in the screening protocol due to budgetary constraints. This limited our ability to assess the proportion of patients with active HDV infection. Second, the study was retrospective and based on data extracted from the MDClone platform, which may be subject to incomplete clinical documentation. Lastly, the study was conducted in a single district in northern Israel, which may limit the generalizability of the findings to other regions or healthcare systems. It is important to note that these limitations may have influenced the study’s outcomes and should be considered when interpreting the results.

Our findings highlight the clinical and public health importance of implementing reflex anti-HDV testing in HBsAg-positive individuals. This strategy improves case identification, may shorten time to diagnosis, and ensures that individuals from high-risk populations, such as those born in endemic regions, are consistently screened. The consistency of our results with the international data supports the integration of reflex testing into standard clinical practice. As effective antiviral treatments become more widely available in the coming years, timely diagnosis will be essential for preventing complications. We believe that our findings provide a strong foundation for developing national screening policies aimed at closing diagnostic gaps and improving outcomes for individuals living with coinfection.

## 5. Conclusions

Anti-HDV reflex testing improved screening coverage, increased detection of anti-HDV seropositive cases, and was associated with shorter time to serologic identification. This approach ensures that all HBsAg-positive individuals are systematically screened, reducing reliance on risk-based strategies that may delay or miss diagnosis. Integration of reflex anti-HDV testing into routine hepatitis B evaluation may support earlier clinical intervention and inform national screening policies aimed at reducing the burden of hepatitis D infection.

## Figures and Tables

**Table 1:** Baseline characteristics of anti-HDV positive patients in the pre-reflex vs. reflex testing cohorts.

Characteristics	Pre-Reflex Cohort	Reflex Testing Cohort	*p*-Value
	*n* = 18	*n* = 21	
Age at HBV diagnosis	48.2 (13.3) [48.2, 41.1–54.5]	53 (15.1) [57, 42.2–61.2]	0.3599
Gender—Female	10 (55.56%)	10 (47.62%)	0.6211
Birth country			0.1140
Israel	11 (61.11%)	10 (47.62%)	
Former Soviet Union	4 (22.22%)	3 (14.29%)	
Ethiopia	1 (5.56%)	6 (28.57%)	
Morocco	2 (11.11%)	0 (0%)	
Else	0 (0%)	2 (9.52%)	
SES			>0.99
High	1 (5.56%)	0 (0%)	
Medium	3 (16.67%)	4 (21.05%)	
Low	14 (77.78%)	15 (78.95%)	
Background disease			
DM	1 (5.56%)	1 (4.76%)	>0.99
HTN	3 (16.67%)	2 (9.52%)	0.6466
CHF	0 (0%)	0 (0%)	-
IHD	1 (5.56%)	0 (0%)	0.4615
CKD	1 (5.56%)	2 (9.52%)	>0.99
HIV	0 (0%)	0 (0%)	-
Laboratory tests			
ALT	28.6 (26.3) [17, 15.5–33]	35.2 (22) [23.5, 18.3–51]	0.1374
AST	30.1 (19.1) [23, 18.7–36]	36.5 (27.8) [25, 20–41]	0.4985
Albumin	3.9 (0.3) [4, 3.9–4.1]	4.1 (0.3) [4.1, 4–4.3]	0.0137
Bilirubin	0.624 (0.208) [0.59, 0.495–0.68]	0.666 (0.23) [0.585, 0.5–0.74]	0.6624
INR	1.021 (0.175) [0.972, 0.9–1.01]	1.023 (0.066) [1.02, 0.98–1.05]	0.2168
PLT	194.5 (63.3) [222.2, 156.4–234.9]	191.6 (55.3) [184, 168.3–219]	0.5686
Creatinine	0.725 (0.109) [0.717, 0.66–0.82]	0.769 (0.144) [0.78, 0.645–0.895]	0.4641

Continuous variables are expressed as mean (standard deviation) [median, interquartile range]. HDV, hepatitis delta virus; SES, socioeconomic status; DM, diabetes mellitus; HTN, hypertension; CHF, congestive heart failure; IHD, ischemic heart disease; CKD, chronic kidney disease; HIV, human immunodeficiency virus; ALT, alanine aminotransferase; AST, aspartate aminotransferase INR, international normalized ratio; and PLT, platelets.

**Table 2:** Comparison of characteristics between anti-HDV positive and negative patients among the HBsAg-positive cohort (percentages of positives by variable).

Characteristics	Anti-HDV-Negative	Anti-HDV-Positive	*p*-Value
	*n* = 853	*n* = 39	
Age at HBV diagnosis	49 (13.3) [47.9, 38.5–58.8]	52.1 (13.9) [54, 42.2–61]	0.1730
Gender			0.3840
Female	377 (95%)	20 (5%)	
Male	476 (96.2%)	19 (3.8%)	
Birth country			0.0691
Israel	602 (96.6%)	21 (3.4%)	
Former Soviet Union	111 (94.1%)	7 (5.9%)	
Ethiopia	60 (89.6%)	7 (10.4%)	
Morocco	38 (95%)	2 (5%)	
Else	40 (95.2%)	2 (4.8%)	
SES			0.8309
High	39 (97.5%)	1 (2.5%)	
Low	611 (95.5%)	29 (4.5%)	
Medium	154 (95.7%)	7 (4.3%)	
DM			0.0234
Yes	136 (99.3%)	1 (0.7%)	
No	717 (95%)	38 (5%)	
HTN			0.1400
Yes	169 (97.7%)	4 (2.3%)	
No	684 (95.1%)	35 (4.9%)	
CHF			>0.99
Yes	15 (100%)	0 (0%)	
No	838 (95.6%)	39 (4.4%)	
IHD			0.7219
Yes	51 (98.1%)	1 (1.9%)	
No	802 (95.5%)	38 (4.5%)	
CKD			0.3049
Yes	103 (98.1%)	2 (1.9%)	
No	750 (95.3%)	37 (4.7%)	
HIV			>0.99
Yes	3 (100%)	0 (0%)	
No	850 (95.6%)	39 (4.4%)	
Period			
Standard testing cohort	550 (96.8%)	18 (3.2%)	0.0200
Reflex testing cohort	303 (93.5%)	21 (6.5%)	

Age was expressed as mean (standard deviation) [median, interquartile range]. HDV, hepatitis delta virus; SES, socioeconomic status; DM, diabetes mellitus; HTN, hypertension; CHF, congestive heart failure; IHD, ischemic heart disease; CKD, chronic kidney disease; and HIV, human immunodeficiency virus.

## Data Availability

The datasets used and/or analyzed during the current study are not publicly available due to data protection regulations but are available from the corresponding author upon reasonable request and with permission from Clalit Health Services.

## Abbreviations

AASLD	American Association for the Study of Liver Diseases
ALT	Alanine aminotransferase
AST	Aspartate aminotransferase
CHF	Congestive heart failure
CKD	Chronic kidney disease
CLIA	Chemiluminescence immunoassay
DM	Diabetes mellitus
EASL	European Association for the Study of the Liver
ECLIA	Electrochemiluminescence immunoassay
HBsAg	Hepatitis B surface antigen
HBV	Hepatitis B virus
HCC	Hepatocellular carcinoma
HDV	Hepatitis D virus
HIV	Human immunodeficiency virus
IHD	Ischemic heart disease
INR	International normalized ratio
IQR	Interquartile range
PCR	Polymerase chain reaction
PLT	Platelets
SES	Socioeconomic status

## References

[1] Puigvehí M., Moctezuma-Velázquez C., Villanueva A., Llovet J.M. (2019). The oncogenic role of hepatitis delta virus in hepatocellular carcinoma. JHEP Rep..

[2] Farci P., Niro G.A. (2012). Clinical Features of Hepatitis D. Semin. Liver Dis..

[3] European Association for the Study of the Liver (2023). EASL Clinical Practice Guidelines on hepatitis delta virus. J. Hepatol..

[4] Miao Z., Zhang S., Ou X., Li S., Ma Z., Wang W., Peppelenbosch M.P., Liu J., Pan Q. (2020). Estimating the Global Prevalence, Disease Progression, and Clinical Outcome of Hepatitis Delta Virus Infection. J. Infect. Dis..

[5] Terrault N.A., Lok A.S.F., McMahon B.J., Chang K.-M., Hwang J.P., Jonas M.M., Brown R.S., Bzowej N.H., Wong J.B. (2018). Update on prevention, diagnosis, and treatment of chronic hepatitis B: AASLD 2018 hepatitis B guidance. Hepatology.

[6] Yurdaydın C., Idilman R., Bozkaya H., Bozdayi A.M. (2010). Natural history and treatment of chronic delta hepatitis: Chronic delta hepatitis. J. Viral Hepat..

[7] Weichselbaum L., Njimi H., Van Den Wijngaert S., Dahma H., Nkuize M., Van Gossum M., Eisendrath P., Mulkay J., Sersté T. (2024). A regular screening for hepatitis delta virus among chronic hepatitis B carriers improves the diagnostic of this infection and of subsequent cirrhosis development. UEG J..

[8] Palom A., Rando-Segura A., Vico J., Pacín B., Vargas E., Barreira-Díaz A., Rodríguez-Frías F., Riveiro-Barciela M., Esteban R., Buti M. (2022). Implementation of anti-HDV reflex testing among HBsAg-positive individuals increases testing for hepatitis D. JHEP Rep..

[9] Nathani R., Leibowitz R., Giri D., Villarroel C., Salman S., Sehmbhi M., Yoon B.H., Dinani A., Weisberg I. (2023). The Delta Delta: Gaps in screening and patient assessment for hepatitis D virus infection. J. Viral Hepat..

[10] Patel E.U., Thio C.L., Boon D., Thomas D.L., Tobian A.A.R. (2019). Prevalence of Hepatitis B and Hepatitis D Virus Infections in the United States, 2011–2016. Clin. Infect. Dis..

[11] Cossiga V., Brusa S., Montalti R., De Conte A., Jannuzzi G., Ranieri L., Sorrentino R., Vallefuoco L., Pignata L., Guarino M. (2024). Anti-HDV reflex testing in HBsAg-positive subjects: An efficacious strategy to identify HDV infection. Liver Int..

[12] Safaie P., Razeghi S., Rouster S.D., Privitera I., Sherman K.E. (2018). Hepatitis D diagnostics: Utilization and testing in the United States. Virus Res..

[13] Eilat-Tsanani S., Zoubi A., Hazzan R. (2022). Detection of Hepatitis D Virus: Performance in the Community. Isr. Med. Assoc. J..

[14] Parfut A., Tripon S., Gantner P., Chaffraix F., Laugel E., Wendling M.-J., Erol F., Wiedemer C., Doffoel M., Saviano A. (2024). Impact of anti-HDV reflex testing at HBs antigen positive discovery in a single center France: Support for primary HDV screening in France. J. Clin. Virol..

[15] Bernhard J., Schwarz M., Balcar L., Hofer B., Dominik N., Strassl R., Aberle S., Munda P., Mandorfer M., Trauner M. (2024). Reflex testing for anti-HDV in HBsAg-positive patients offers high diagnostic yield in a large Central European tertiary care center. Sci. Rep..

[16] John B.V., Bastaich D., Amoli M.M., Wong R.J., Evon D.M., Rogal S.S., Ross D.B., Morgan T.R., Spector S.A., Villada G. (2024). Association of HDV infection and HCC, hepatic decompensation, and all-cause and liver-related death in a national cohort. Hepatology.

[17] Cai C., Zeng J., Wu H., Shi R., Wei M., Gao Y., Ma W. (2015). Association between hepatitis B virus infection and diabetes mellitus: A meta-analysis. Exp. Ther. Med..

[18] Huang S., Kao J. (2024). The interplay between chronic hepatitis B and diabetes mellitus: A narrative and concise review. Kaohsiung J. Med. Sci..

